# Deep Learning-Based 6-DoF Object Pose Estimation Considering Synthetic Dataset

**DOI:** 10.3390/s23249854

**Published:** 2023-12-15

**Authors:** Tianyu Zheng, Chunyan Zhang, Shengwen Zhang, Yanyan Wang

**Affiliations:** School of Mechanical Engineer, Jiangsu University of Science and Technology, Zhenjiang 212100, China; 211110201122@stu.just.edu.cn (T.Z.); justzhang2003@126.com (S.Z.); 211210201101@stu.just.edu.cn (Y.W.)

**Keywords:** 6-DoF object pose estimation, synthetic dataset, deep learning, bilateral filtering, CBAM–CDAE

## Abstract

Due to the difficulty in generating a 6-Degree-of-Freedom (6-DoF) object pose estimation dataset, and the existence of domain gaps between synthetic and real data, existing pose estimation methods face challenges in improving accuracy and generalization. This paper proposes a methodology that employs higher quality datasets and deep learning-based methods to reduce the problem of domain gaps between synthetic and real data and enhance the accuracy of pose estimation. The high-quality dataset is obtained from Blenderproc and it is innovatively processed using bilateral filtering to reduce the gap. A novel attention-based mask region-based convolutional neural network (R-CNN) is proposed to reduce the computation cost and improve the model detection accuracy. Meanwhile, an improved feature pyramidal network (iFPN) is achieved by adding a layer of bottom-up paths to extract the internalization of features of the underlying layer. Consequently, a novel convolutional block attention module–convolutional denoising autoencoder (CBAM–CDAE) network is proposed by presenting channel attention and spatial attention mechanisms to improve the ability of AE to extract images’ features. Finally, an accurate 6-DoF object pose is obtained through pose refinement. The proposed approach is compared to other models using the T-LESS and LineMOD datasets. Comparison results demonstrate the proposed approach outperforms the other estimation models.

## 1. Introduction

In recent years, the 6-Degree-of-Freedom (DoF) object pose estimation has garnered significant attention in the field of computer vision and robotics, owing to its relevance in several applications such as robot grasping, active driving, and augmented reality. The task involves estimating the orientation and the pose of a target object in 3D space. While traditional 6-DoF object pose estimation methods can produce satisfactory results in simple scenes, a growing number of researchers are currently dedicating their efforts to exploring methods and tasks using deep learning (DL)-based approaches.

Advances in DL techniques have led to significant progress not only in the areas of target detection [1,2,3] and image segmentation [4,5,6,7,8,9,10,11], but also significant progress has been made in pose estimation using these techniques. They can be classified based on the types of datasets into (1) approaches relying on real datasets [12,13,14,15,16,17,18,19,20,21,22,23]; and (2) approaches based on synthetic data [24,25,26,27,28,29,30,31,32]. However, the need for labeled real datasets raises a challenge due to the time-consuming and labor-intensive nature of their production, resulting in high dataset production costs [33]. To address the scarcity of real data, the researchers have proposed approaches based on synthetic data. Nonetheless, a gap resides between synthetic and real data, making it challenging to apply networks trained using synthetic data when considering real environments. To bridge this gap, some researchers have proposed some excellent 6-DoF position estimation methods for objects relying on synthetic data. Some classical methods for 6-DoF position estimation based on synthetic data are presented below. For instance, Yu et al. [24] utilized Blender to generate a high-fidelity large-scale synthetic dataset of objects and designed a TGF-Net network for feature learning to achieve object 6-DoF position estimation. Sundermeyer et al. [25] introduced the augmented autoencoder (AAE) to implicitly represent pose features, reducing the domain gap between synthetic data and real data through the domain randomization (DR) method. They also proposed sharing a separate self-coder for each object in a multi-object scenario [26]. Furthermore, the proposed 6IMPOSE [27] framework validates the efficiency of the position estimation model acquired from synthetic data training in real scenes. However, the accuracy of the object 6-DoF position estimation method, based on synthetic data, is notably lower than that based on real data on the datasets [34,35]. To make progress in detection accuracy, Huang et al. [30] proposed a network based on variant AAE network [25]. Moreover, with the help of a textureless CAD model and a small number of real images, some researchers [29,32] improved detection accuracy through patch-level realism images.

Despite these methods having attempted to mitigate the domain gap between synthetic data and real data, however, it still exhibits a domain gap between real data and synthetic data, leading to a shortfall in achieving satisfactory accuracy due to the model limitations. Additionally, most methods struggle to deliver good results when dealing with special surfaces, such as weak textures and occlusions. Therefore, this paper aims to explore a high-precision object 6-DoF position estimation method based on synthetic data and DL. Moreover, in this paper, to enhance the robustness, scalability, and flexibility of the network, the 6-DoF pose estimation network for objects was decoupled to include: (1) an instance segmentation network incorporating target detection and image segmentation; (2) a latent feature extraction network based on unsupervised learning.

To sum up, this paper primarily focuses on the systematic optimization of synthetic data and network structures to enhance the 6-DoF detection accuracy, particularly for weakly textured objects based on synthetic data.

- Regarding the dataset, images of real realistic 3D object models are used. Specifically, in segmentation network detection, incorporating images of these real 3D object models proves effective in significantly and greatly reducing the domain gap between the synthesized data and the real data [36]. Therefore, more realistic and clearer details are obtained by performing bilateral filtering and image sharpening operations on the obtained images in the synthetic dataset.
- Regarding the network, this paper focuses on the improved mask region-based convolutional neural network (R-CNN) [37] and the improved convolutional denoising autoencoder (CDAE) networks. In the instance segmentation network part, to improve the accuracy and speed of target detection and image segmentation, an M-ST instance segmentation network, namely Mask Swin Transformer (M-ST) is designed. The idea of the self-attention mechanism [38] is encompassed into the Mask R-CNN network, incorporating the window polytope self-attention module and displacement window self-attention module, to enhance detection accuracy while significantly reducing model computation. Simultaneously, we add a layer of the bottom-up path to the original FPN structure to obtain the iFPN structure, addressing the shortcoming of the traditional FPN structure where the input feature map only contains the feature information of the current layer and the previous layer, lacking the internalization of the underlying features. As for the improved CDAE network, the DR method for training the CDAE is applied [24]. Moreover, this trained network is used as a pose feature extraction network, generating templates of various poses in virtual space to create a codebook for subsequent template-matching tasks. To enhance the network’s feature extraction capability, and generalization ability, and improve convergence and training efficiency, a convolutional block attention module (CBAM) [39] is integrated with an attention mechanism module, added to the CDAE network.
- Finally, the object bounding box information obtained from the M-ST network is used to crop the image into the trained CBAM–CDAE network for template matching to obtain the initial 6-DoF bit pose. Using the iterative closest point (ICP) algorithm, the object segmentation map obtained from the example segmentation algorithm is combined with the object point cloud generated from the depth image to achieve precise alignment of the position.

## 2. Related Works

In this section, to have a comprehensive understanding of the object 6-DoF methods, common techniques for target detection and segmentation are first introduced; then, 6-DoF object methods, based on real and synthetic data, are presented.

### 2.1. Object Detection and Image Segmentation Methods

Object detection methods can be sparsed into two categories: single-stage object detection algorithms [1,2] and two-stage object detection algorithms [3]. The former category includes You Only Look Once (YOLO) [1] and Single Shot Detector (SSD) [2] algorithms. In more detail, YOLO was first proposed by Redmon et al. [1], directly predicting bounding box and category probabilities in a single forward pass by means of regression and achieving, as a result, real-time target detection. In 2016, Liu et al. introduced the SSD algorithm [2], utilizing feature maps at multiple scales and multiple convolutional layers with the aid of anchor frames. This approach significantly improved detectability and target detection accuracy.

They ensure real-time detection but encounter lower performance compared to the two-stage algorithms. In the two-stage algorithms, Faster R-CNN [3] is a representative two-stage target detection algorithm, known for its high localization and recognition accuracy. Faster R-CNN [3] consists of the region proposal network (RPN) in the first stage, generating candidate object bounding boxes. As for the second stage, features are extracted from each candidate box using the RoI layer to perform classification and bounding box regression tasks. 

Furthermore, instance segmentation is a computer vision task derived from target detection and semantic segmentation. Moreover, instance segmentation methods can be categorized as single-stage or two-stage techniques. 

In more detail, single-stage methods include the You Only Look At Coefficient Ts (YOLACT) technique [4], which is an algorithm based on the RetinaNet network [5], the Mask-IoU loss function was applied firstly using the YOLACT algorithm to optimize target segmentation and mask prediction, exhibiting faster processing speed and higher accuracy. The Segmenting Objects by LOcations (SOLO) instance segmentation algorithm, proposed by Wang et al. [6], transformed the task of target segmentation into predicting the position of the target instances and the segmentation mask. The segmentation of target instances was directly performed at each pixel point, avoiding the need for anchor frames. TensorMask [7] provided an effective solution for the target segmentation task by introducing new architectures and technical means such as loss functions to improve the segmentation performance.

Regarding the two-stage instance segmentation algorithms, they can be divided into top-down and bottom-up approaches. Representative algorithms in this category include a fully convolutional instance-aware semantic segmentation (FCIS) [8] and Mask R-CNN, FCIS was an instance semantic segmentation model based on fully convolutional networks (FCNs) that could predict the entire image directly, eliminating the need for anchor frames and thereby improving segmentation accuracy and speed. Mask R-CNN, on the other hand, extends a segmentation (Mask) branch to Faster R-CNN and introduces a RoIAlign layer. This layer ensures more accurate feature extraction within each candidate region, facilitating pixel-level segmentation prediction. This achieves excellent performance for target detection and instance segmentation tasks. In addition, due to the effectiveness of the Mask R-CNN method, a large number of related algorithms have been derived from it [9,10]. It is worth noting that bottom-up methods [11] typically have lower effectiveness compared to top-down methods.

In this paper, to balance the recognition accuracy and the recognition speed, we first adopt the two-stage detection algorithm, using the Mask R-CNN as the base instance segmentation model. Consequently, we simplify the model by replacing the structure of the backbone network and higher quality synthesized data, which would further improve the detection accuracy through the modification of the internal structure.

### 2.2. A 6-DoF Object Pose Estimation Methods

#### 2.2.1. A 6-DoF Object Pose Estimation Methods Based on Real Datasets

Traditional 6-DoF object attitude estimation methods frequently solve simple object position estimation tasks. Therefore, these methods are not sufficient to face the increasing industrial demands. However, with the rapid development of DL, the traditional 6-DoF object pose estimation is evolving towards the use of DL-based 6-DoF object pose estimation methods. In more detail, DL-based 6-DoF pose estimation methods can be classified into two categories: pose regression-based and key point-based methods. Depending on the dataset, these methods can be further categorized as real data-based or synthetic data-based methods. Going back to the first classification, the pose regression-based method is a commonly used approach for 6-DoF pose estimation, where the 6D pose of the object is directly estimated by this method. Among the different methods, the most used one is the SSD-6D [12], which extends the 2D target detection network SSD [2] to a 3D detection and a 3D rotation estimation. Moreover, this technique is more accurate for object pose estimation with rich features; however, it is less effective for recognizing weakly textured or non-textured objects. In addition, the SSD-6D is not an end-to-end network.

As for the coordinates-based disentangled pose network (CDPN) network approach [13], proposed by Li et al., it learns the pose parameters of the target object directly from the image. It is characterized by its simple and compact network structure with a reduced number of parameters, allowing it to achieve fast inference with limited computational resources. For instance, Jin et al. [14] introduced a translation module to enable initial translation of the depth map, and used a pose regression module to combine the RoI and the original image to predict the rotation and optimize the translation, achieving better results; however, the main drawback was at the level of the detection speed.

The key point-based approach applies a two-stage strategy instead of directly predicting the 6D object pose. Through this approach, the first stage involves detecting the 2D key points of the target object when the complete 3D model of the target is known. Then, the Perspective-n-Point (PnP) algorithm is employed to calculate the 6-DOF pose based on the correspondence between the 2D and 3D key points. Among the different methods, the BB8 [15] algorithm presents a two-stage pose estimation framework, the 6-DoF pose of the object was predicted with the 2D projection of the eight vertices of a 3D bounding box combined with the PnP algorithm. To address the ill-posedness problem in pose estimation for various types of rotational symmetry, the rotation range of the training image was restricted, effectively resolving the issue. While YOLO-6D [16] is an end-to-end model built based on the YOLO base framework, the core idea was to transform the position estimation task into a target detection problem. For instance, Vidal et al. [17] employed improved point-to-point features for 6-DoF position estimation. The algorithms proposed by BB8 [15], YOLO-6D [16], and Vidal et al. [17] lead to excellent results when solving the 6D pose based on key points. Moreover, BB8 and YOLO-6D are applied in the LINEMOD dataset [34], whereas the method proposed by Vidal et al. demonstrates excellent performance when applied to the T-LESS dataset [35].

However, the main drawback of these methods is their susceptibility to occlusion and noise due to their heavy reliance on global information. This led to the development of a series of excellent 6-DoF pose estimation methods, based on pixel voting. For instance, ZAKHAROV et al. [18] proposed a 3D object detection and pose estimation method, employing three encoders to obtain U, V, and ID masks. By combining the ID mask with the 3D object model and the 2D–3D point pairs obtained from the UV combination, the PnP algorithm is applied. For example, Peng et al. developed the pixel-wise voting network (PVNet) [19] network, combining 3D shape information and 2D projection information for pose estimation. However, the network was trained separately for each class, limiting its ability to detect multiple objects of different classes simultaneously. Moreover, Vidal et al. [20] applied the top-down visual attention and color cues technique to improve the performance of state-of-the-art methods in occluded scenes. The obtained results showed excellent performance on public datasets. In addition, a deep point-wise 3D key points voting network (PVN3D) [21] proposed a deep Hough voting network to detect the 3D key points of an object and then estimate the 6D pose parameters using a least squares fitting approach. Added to that, Pix2pose [22] consisted of a pose estimation method for weakly textured or non-textured objects. It applies RGB images to predict the 3D coordinates of each object and the expected variance at the level of each pixel. The 2D–3D correspondence is then established using pixel-by-pixel prediction in multiple stages, enabling direct prediction of 6D pose based on PnP and RANSAC. Furthermore, to address occlusion problems, the Pix2pose method employs a generative adversarial network (GAN) to recover the occluded regions. Moreover, it introduces a new loss function, known as the transformer loss for 3D coordinate regression, helping in resolving object symmetry issues. Meanwhile, Hajari et al. [23] proposed a method, based on point cloud template matching, to realize some progress in position estimation of weakly textured objects. Within the pose estimation task, it is challenging to cover all object poses during training by just using real data; thus, acquiring pose labels with ground truth values is difficult to realize in several scenarios.

#### 2.2.2. A 6-DoF Object Pose Estimation Methods Based on Synthetic Datasets

As real datasets often have limited generalization ability, methods have been developed for estimating the 6D pose of objects based on synthetic data. For example, a convolutional network PoseNet [28] applies a Leigh CNN to localize objects in real images and trains them based on synthetic single-channel images to directly regress the 6D pose of objects in real images with no need for additional engineering or graph optimization, it can operate indoors and outdoors in real time, taking 5 ms per frame to compute. Moreover, Marion et al. [29] proposed transferring the domain where the synthetic and real images are located to the pencil filter domain in order to increase the visual similarity in the new domain. As for the adversarial autoencoder (AAE) [25], it has used 3D object models to synthesize data instead of annotating data for training. It also introduced the concept of computing global descriptors of localized object instances applying an AAE network. Using excluding noise, this method tried to solve the challenges regarding the domain gap between real and synthetic data. However, test results, performed on some datasets, showed that the accuracy of the 6D localized pose estimation for target objects is not satisfactory. Therefore, Huang et al. [30] proposed a network based on a variant AAE, achieving a certain improvement in accuracy compared to the CDAE-based method of the AAE network. Moreover, SyDPose [31] used synthetic depth data with neighborhood-correlated background random noise heuristics to train end-to-end multitasking networks to perform the pose estimation task. In addition, Xu et al. [32] introduced an image-to-image translation-based synthetic data generation method, requiring only texture-free CAD models and a small number of real images. The proposed method demonstrated relatively excellent results on the T-LESS dataset. Furthermore, to solve the problem of poor generalization ability of synthetic data- based object 6-DoF pose estimation for practical applications, the 6IMPOSE [27] technique overcame the shortcomings of the PVN3D [21] algorithm in terms of generalization and performance, achieving better results in synthetic data-based pose estimation algorithms and confirming the validity of the synthetic data developed for real scenes.

However, the limited research regarding depth information leads to certain limitations. In order to reduce the domain gap of 6-DoF object-positioning-based methods, as well as to solve the problem of low accuracy, the following methods are innovatively proposed in this work.

So, to reduce the domain gap between the synthetic data and the real one, higher quality synthetic datasets are created with the objective of detecting and instantly segmenting stages. In more detail, the idea of the AAE network is mimicked and adopted to the DR method to reduce the domain gap problem during the training process. As for the shortcomings of the Mask R-CNN network (having mainly a large number of network parameters), it was replaced by the Swin Transformer backbone network. Moreover, the original FPN structure was modified, leading to a reduction in the overall network parameters and significantly improving the recognition accuracy. Meanwhile, regarding the CDAE network, the idea of an attention mechanism was incorporated into the CBAM module; therefore, the whole network achieved better results. Finally, the details will be presented in Section 3.

## 3. Methodology

In this section, the general framework of the network, developed in this paper, is first introduced. Then, the generation of high-quality synthetic datasets involved in this work is introduced. Finally, the M-ST instance segmentation network, the CBAM–CDAE network, and the pose refinement method are detailed.

### 3.1. Framework of Proposed Object 6-DoF Pose Estimation Method

**Figure 1:**
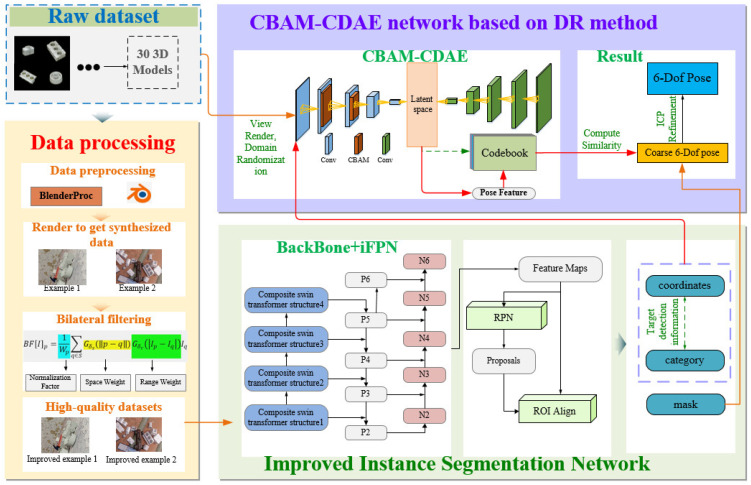
Framework of the methodology proposed in this paper.

In this paper, our method consists of estimating the 6-DoF pose of an object based on a single RGB/RGB-D image. This method’s general framework is displayed in Figure 1. The method is divided into modules, such as high-quality synthetic data processing, image instance segmentation, and feature extraction networks based on unsupervised networks. By incorporating bilateral filtering techniques to obtain high-quality synthetic data, the domain gap between synthetic and real data will be narrower. In addition, to reduce the computational cost and improve the accuracy of the detection model, a novel attention-based Mask R-CNN network is proposed. However, an iFPN is developed by adding a layer to bottom-up paths to extract the underlying internalized features. Consequently, a novel CBAM–CDAE network is proposed to enhance the ability of the AE to extract image features by introducing channel attention and spatial attention mechanisms. Finally, at each instance, the relation to the feature extraction network of the unsupervised network is computed to generate a code set, the initial pose is estimated using a template matching method, and the final object pose is acquired through refinement.

### 3.2. Higher Quality Dataset Based on BlenderProc and Bilateral Filtering

**Figure 2:**
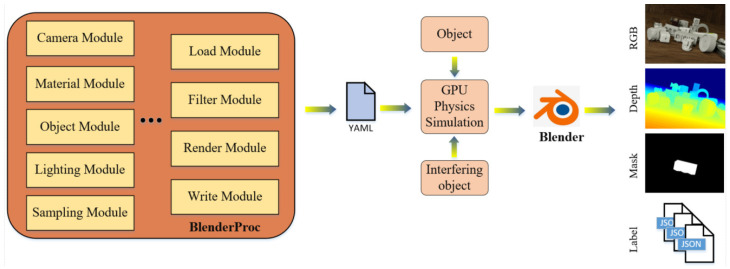
BlenderProc-based synthesized data production process.

In this paper, the M-ST instance segmentation network serves as a target detection and segmentation network based on synthetic data. However, the high-quality PBR method is an important step in the processing stage of synthetic data. It plays a key role in improving target detection accuracy and reducing the domain gap between synthetic and real data [36,40]. The initial dataset in this paper comprises reduced versions from BOP [41] including LineMOD and T-LESS datasets (specific characteristics of these datasets in question are described in Section 4). These datasets were generated by BlenderProc. BlenderProc is a modular program pipeline based on Blender, enabling the synthesis of training images with high visual realism and the customization of a variety of annotation information, such as mask, depth, and 6-DoF pose, catering to a wide range of computer vision tasks. BlenderProc includes several modules such as a camera module, object module, material module, and lighting module, among others, providing flexibility according to the task requirements to write configuration files, and import the 3D model into GPU for physical simulation. The common rendering process of BlenderProc is illustrated in Figure 2.

To achieve higher quality synthesized images, we found that applying bilateral filtering to the synthesized image enhances edge information and image details, resulting in a clearer image and the removal or attenuation of noise. Bilateral filtering considers the spatial relationship between pixels, selecting a domain range of pixels to be processed for each pixel. This limitation in the processing range allows bilateral filtering to retain local details of the image.

**Figure 3:**
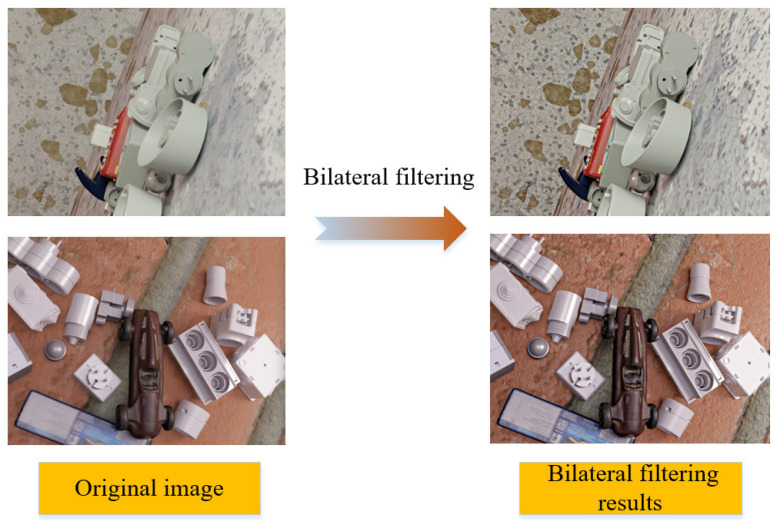
Results of bilateral filtering.

Moreover, bilateral filtering considers the grayscale difference between pixels. The grayscale difference serves as a weighting coefficient, multiplied by the pixel value, and then applied as a weighted average to each pixel. This weighted average operation effectively preserves edge information in the image. An example is illustrated in Figure 3.

The use of bilateral filtering for image sharpening, in contrast to other methods, such as Laplace operator-based image sharpening, does not generate additional noise. Therefore, bilateral filtering can achieve better results in the processing of synthetic images. Finally, we further enhance the images by randomly adjusting contrast, and saturation, and introducing random Gaussian noise and blur to increase the diversity of the training set.

### 3.3. M-ST Instance Segmentation Network

In this paper, the aim is to increase the speed and accuracy of model recognition, thereby improving the level of 6-DoF object pose estimation. The substitution of the Mask R-CNN backbone network with Swin Transformer reduces the model parameters, leading to increased model recognition accuracy. Simultaneously, to address the deficiency of underlying feature information in the input feature maps within the traditional FPN structure, an additional layer of bottom-up structure is added, resulting in an improved FPN structure.

The Swin Transformer, introduced in 2021 by researchers from Microsoft Research, led by Han Hu, has emerged as a replacement for the traditional CNN architecture, showcasing superior performance. Despite its transformative impact on computer vision, the Swin Transformer, as a model based on the Transformer architecture, has not garnered much attention for industrial applications. To explore its scalability, we endeavor to extend the applicability by combining Swin Transformer and Mask R-CNN implementations and applying them to our specific task.

The M-ST network introduced in this paper builds upon the Mask R-CNN network, which, in turn, is an improvement of the Faster R-CNN algorithm. The Mask R-CNN algorithm is designed to conduct both target detection and semantic segmentation. It achieves this by utilizing the RoI obtained through RoIAlign and incorporating a parallel Mask branch.

**Figure 4:**
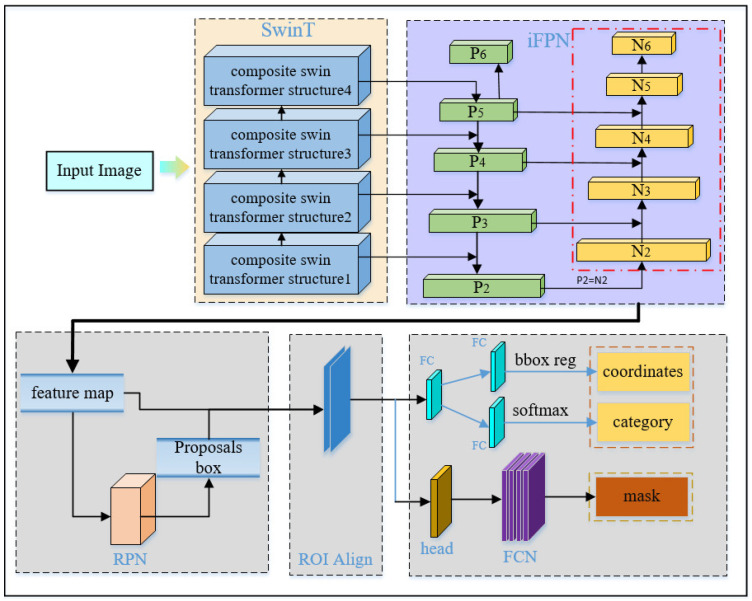
M-ST network structure.

Referring to Figure 4, in the backbone network, composite structure 1 consists of a patch merging layer along with a linear embedding layer and Swin Transformer block, whereas composite structures 2 to 4 consist of a patch merging layer combined with Swin Transformer block structure.

To achieve pixel-to-pixel predictive masking and multi-scale feature fusion, Mask R-CNN employs a feature pyramid network to obtain deeper feature information. However, the extended fusion path between low-level features and high-level features results in the underutilization of low-level feature location information, impacting semantic segmentation accuracy. In contrast, the Swin Transformer introduces multi-scale feature modeling, a local window mechanism, and a sliding window operation to enhance model recognition efficiency while reducing computational complexity. Specifically, by incorporating the patch merging layer and Swin Transformer block in the Mask R-CNN network, the network focus is shifted to the interaction of cross-scale information. This allows effective capture of semantic and contextual information in images at different scales, achieving improved scale complexity simultaneously.

1. Patch Merging layer

**Figure 5:**
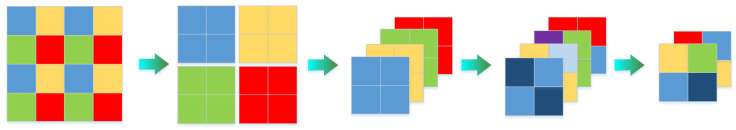
Patch merging layer schematic.

The slice merging layer, as previously described, plays a crucial role in downsampling the feature map. However, in the context of the composite Swin Transformer structure1, a patch merging layer is employed to downsample the feature map, as illustrated in Figure 5. The resulting four feature maps are then concatenated along the depth direction and passed through a LayerNorm layer. To conclude, the depth of the feature map undergoes a linear transformation from C to C/2 through a fully connected layer.

Compared to traditional pooling or convolutional layers that necessitate a large number of parameters for downsampling, the patch merging layer achieves downsampling by directly integrating features at multiple small spatial locations. It is worth noting that this is achieved without adding extra parameters or computation load. Due to its capability to retain more spatial information, the patch merging layer improves the model’s ability to recognize object size and shape. Furthermore, the patch merging layer contributes to a richer feature map resolution by merging different paths in each iteration.

2. Swin Transformer block

**Figure 6:**
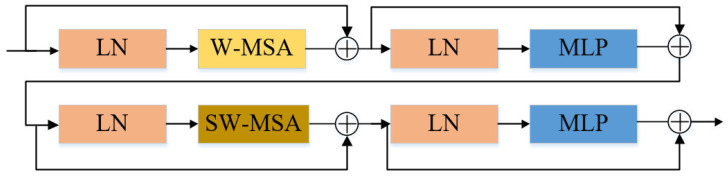
Swin Transformer block.

The Swin Transformer layer consists of a normalized layer (LayerNorm), Windows Multi-head Self Attention (W-MSA), Shifted Windows Multi-head Self Attention (SW-MSA), and Multi-Layer Perceptron (MLP), as illustrated in Figure 6.

In this paper, the M-ST network employs different MSA blocks, namely the W-MSA and SW-MSA. W-MSA divides the input image into non-overlapping windows, each containing multiple patches, and then computes self-attention within these windows. This approach significantly reduces the complexity of the self-attention calculation, enhancing interaction between different locations and improving feature representation capability. However, due to the lack of information interaction between windows, extracting high-level semantic information from the image becomes challenging. To overcome this limitation, the SW-MSA module is introduced, connecting adjacent but non-overlapping windows in the upper layer. The design increases the perceptual field and captures higher level semantic information. Therefore, to alternate between the W-SMA module and the SW-MSA module, two or a multiple of two Swin Transformer blocks are used consecutively, as shown in Figure 6. 

The self-attentive operation serves as the core of the Swin Transformer layer. Initially, the input feature map is linearly transformed into a two-dimensional sequence dataset. Subsequently, *Q*, *K*, and *V* are computed using a fully connected layer. These *Q*, *K*, and *V* values are then input into the proportional dot product attention component for processing. The results are spliced, and finally fed into a fully connected layer to obtain the final result, as shown in Equation (1):

(1) $$S=softmax\left(\frac{QK^{T}}{\sqrt{d_{k}}}\right)V$$

where *S* represents the self-attentive operation, *d* denotes the dimensionality set of the model, and, finally, *Q*, *K*, and *V* indicate the values of the linear transformation of the feature map.

The multi-layer perceptron is responsible for classifying the category information of the input feature map. Comprising two fully connected layers, an activation function layer using the Gaussian error linear unit (GELU) function, and two random deactivation layers, the multi-layer perception plays a crucial role in preventing model overfitting.

3. The iFPN structure

The feature maps input to the RPN within the FPN structure include only the feature information of the current and upper layers, lacking details from the lower layers. However, the feature maps of the bottom layers contain more detailed information. To address this, in this paper, we introduce channels that connect from the bottom to the top and then backward. The iFPN structure is depicted in Figure 1, where $P_{i}$ (*i* = 2, 3, 4, 5, 6) represents the feature pyramid, and the newly added bottom-up path merges the low-level feature map *N* with the high-level feature map *P* to generate a new feature map *N*.

### 3.4. CBAM–CDAE Network

In this paper, the concept of applying the DR technique to train on simulated views of a 3D model was inspired by AAE network. Furthermore, an almost positive polyhedral triangle substitution method was implemented to ensure sampling from a sufficiently homogeneous viewpoint of the virtual camera. However, the trained pose feature extraction network often produces potential vector representations that lack accuracy. To enhance recognition accuracy and improve the extraction capability of the traditional CDAE network, CBAM was incorporated into CDAE.

#### 3.4.1. Uniform Multi-Viewpoint Generation

**Figure 7:**
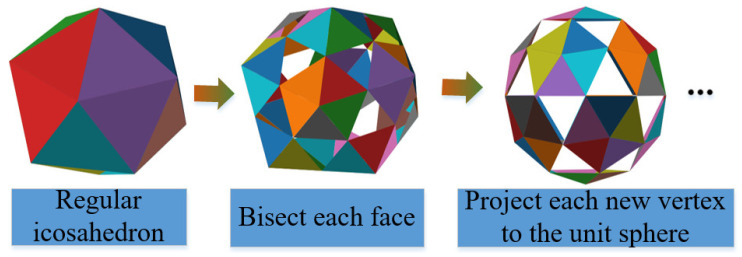
Isometric sampling based on ortho-20 facets.

To acquire template images uniformly distributed in SO(3) space, as illustrated in Figure 7, the sampling process entails placing the virtual camera at viewpoints where the vertices of the almost ortho-polyhedron are sampled from the recursive triangular decomposition of the hemisphere above the object.

#### 3.4.2. CBAM–CDAE Network Structure

To overcome the limited feature extraction capability of AAE networks, this paper introduces CBAM–CDAE networks that incorporate CBAM and are trained on simulated views using DR. The original AE is an unsupervised model, with the encoder mapping input data to a low-dimensional latent space and the decoder reconstructing the latent representation to the original data after upsampling. 

However, the encoded values output by the encoder encompass various information such as category, pose, and displacement, making it challenging to represent individual pose features. In contrast, CDAE excels at extracting valuable features while filtering out noise. By treating all information other than pose as noise, the encoded values output by the encoder can effectively represent pose-related features.

Introducing the CBAM module enhances CDAE’s ability to capture channel correlation and spatial correlation in the input data. This improvement significantly enhances feature extraction, particularly for weakly textured objects. The structure of CDAE can be expressed using Equation (2):

(2) $$\tilde{x}=\left(\gamma \cdot \varphi \cdot f_{noise}\right)\left(x\right)=\gamma \left(z\right)$$

where *x* represents the original input data, *z* denotes the encoded value, and $\tilde{x}$ indicates the reconstructed data. Moreover, the encoded value *z* is an implicit representation of the original input data *x*, whose dimensionality is usually lower than *x* and $\tilde{x}$. In addition, the $f_{noise}$ represents the additional noise, whereas parameters related to background addition, changing image contrast, height, Gaussian blur and color distortion, and random black square occlusion are derived from the AAE network.

**Figure 8:**
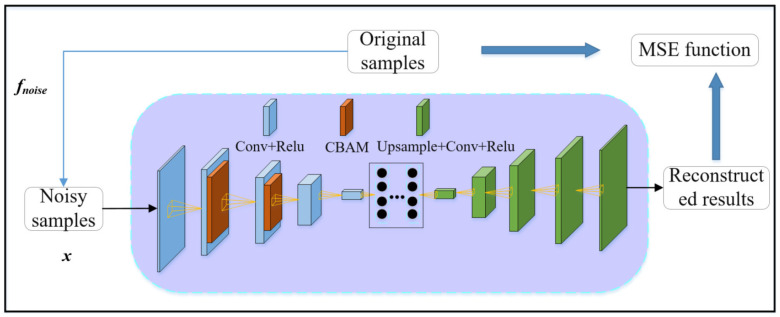
CBAM–CDAE network structure.

The CBAM–CDAE network structure is illustrated in Figure 8.

**Figure 9:**
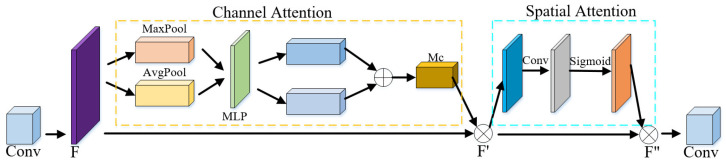
CBAM network structure.

The convolutional attention module CBAM is displayed in Figure 9.

In 2018, Woo et al. [39] proposed the CBAM technique, using a combination of channel attention and spatial attention algorithms to generate an attention graph. This approach allows the network to focus on important local details while filtering out unimportant local information. Moreover, this learning method highlights local key features, suppresses irrelevant features, and enhances the network’s ability to express features. 

Referring to Figure 8, this paper splices the CBAM network after the second and third convolutional layers of CDAE. This not only enhances the feature learning capability of the network by channel and spatial learning of image features and enables the encoder to put more attention on important local features and filter out unimportant local features, but it also prevents overfitting in training due to the overly complicated model CBAM consisting of a channel attention module and a spatial attention module, as displayed in Figure 9.

The details about the way of work of CBAM are determined as follows. The channel attention module calculates the global maximum pooling and the global average pooling of the input feature map *F*, resulting in two *1 × 1 × N* feature vectors. These vectors are then fed into the multilayer perceptron. The output of the perceptron is added to the two feature vectors, and the result is activated using sigmoid to produce the channel attention matrix. Multiplying the channel attention matrix with the input feature map *F* yields the channel attention module’s output feature map $F^{\prime }$. The computational representation of the channel attention module is as follows:

(3) $$M_{c}\left(F\right)=\delta \left(MLP\left(AvgPool\left(F\right)\right)+MLP\left(MaxPool\left(F\right)\right)\right)$$

(4) $$F^{\prime }=M_{c}\left(F\right)\times F$$

In Equations (3) and (4), F represents the feature map, *MLP* denotes the multilayer perceptron, *AvgPool* and *MaxPool* indicate the average pooling and maximum pooling, respectively, $M_{c}$ represents the channel attention matrix, and, finally, $F^{\prime }$ is the output feature of the channel attention module.

The spatial attention module initially applies maximum and average pooling on the feature map in the channel dimension, resulting in two *W × H × 1* feature maps. These are then concatenated into a *W × H × 2* feature map, which undergoes further processing using convolutional layers to extract features, ultimately obtaining a *W × H × 1* feature map. The sigmoid activation function generates the spatial attention feature matrix, representing the weights of the channels occupied by each pixel. Multiplying this matrix with the feature map $F^{\prime }$ yields the spatial attention feature map $F^{\prime \prime }$. The feature map $F^{\prime \prime }$ and the input feature map F are added to obtain the input for the next convolutional layer. Finally, the calculation of the spatial attention module is expressed as follows:

(5) $$M_{s}\left(F^{\prime }\right)=\delta \left(f\left[AvgPool\left(F^{\prime }\right);MaxPool\left(F\right)\right]\right)$$

(6) $$F^{\prime \prime }=M_{s}\left(F^{\prime }\right)\times F^{\prime }$$

In the above expressions, *f* represents the convolutional layer and denotes the serial connection, $M_{s}$ is the spatial attention feature matrix, and $F^{\prime \prime }$ indicates the feature map generated by the spatial attention module.

Among them, the pooling layer plays a crucial role in subsampling, aiming to reduce dimensionality, mitigate overfitting, and improve the robustness of the network. Moreover, the common types of pooling include maximum and average pooling. In this paper, the inclusion of maximum pooling serves to decrease the computational complexity of the upper layer by eliminating non-maximum values while providing translation invariance. 

### 3.5. The 6-DoF Object Pose Decoupling Calculations

#### 3.5.1. Template Matching Based 3-DoF Object Rotation Pose

In this paper, the bounding box information obtained from the M-ST instance segmentation network is used to crop the image, adjusting the input size based on the longer side of the bounding box multiplied by a fill factor of 1.2. This cropped image is then fed into the pose feature extraction network to obtain the pose feature *z_test* for the object image. To expedite template matching, cosine similarity is employed to measure the feature similarity between the object image and the template one. Moreover, the cosine similarity is expressed as follows:

(7) $${similarity}_{i}=\frac{z_{i}z_{test}}{\left\|z_{i}\right\|\left\|z_{test}\right\|}$$

Following the calculation of similarity between the object image and template images from the codebook information, the K-nearest neighbor (KNN) algorithm is applied to identify the *k* templates closest to the pose of the object image. The pose *R* corresponding to these *k* templates serves as the hypothetical pose of the object image.

#### 3.5.2. Calculation of 3-DoF Object Translation Pose

In this paper, the M-ST network provides the bounding box information of the object $\left(u_{a},v_{a},w_{a},h_{a}\right)$. Additionally, after estimating the object’s rotation, the bounding box information of the object in the nearest neighboring template image $\left(u_{b},v_{b},w_{b},h_{b}\right)$ can be obtained. Therefore, leveraging the imaging model of the camera, we can derive:

(8) $$\frac{t_{a,z}\cdot \sqrt{w_{a}^{2}+h_{a}^{2}}}{f_{a}}=\frac{t_{b,z}\cdot \sqrt{w_{b}^{2}+h_{b}^{2}}}{f_{b}}$$

where $t_{a,z}$ represents the *z*-axis displacement of the object, $t_{b,z}$ indicates the *z*-axis displacement of the object in the template image, and $f_{a}$ and $f_{b}$ denote, respectively, the focal length of the true and rendered cameras. Then, the estimated value of $t_{a,z}$ is formulated as follows:

(9) $$t_{a,z}=t_{b,z}\cdot \frac{f_{a}}{f_{b}}\cdot \frac{\sqrt{w_{b}^{2}+h_{b}^{2}}}{\sqrt{w_{a}^{2}+h_{a}^{2}}}$$

To solve for the displacements $t_{a,z}$ and $t_{a,y}$ in the image plane coordinate system of the object on the *x*-axes and *y*-axes, it is necessary to estimate first the pose of the object center on the image plane ($x_{a}$, $y_{a})$:

(10) $$\left\{\begin{array}{c} x_{a}=u_{a}+\frac{w_{a}}{2}-c_{x,a}\\ y_{a}=v_{a}+\frac{h_{a}}{2}-c_{y,a} \end{array}\right.$$

where $c_{x,a}$ and $c_{y,a}$ represent the offset of the image plane coordinates. Similarly, the pose of the object center in the template image on the image plane ${(x}_{b},y_{b})$ can be determined. Moreover, the transformation relationship between the image and the camera coordinate systems is denoted as follows:

(11) $$\left\{\begin{array}{c} x_{a}=\frac{f_{x,a}\cdot t_{a,x}}{t_{a,z}}\\ y_{a}=\frac{f_{y,a}\cdot t_{a,y}}{t_{a,z}} \end{array}\right.$$

The displacements of the object on the *x*-axes and *y*-axes can be obtained according to Equations (10) and (11), which are expressed as follows:

(12) $$\left\{\begin{array}{c} t_{a,x}=\frac{x_{a}}{f_{x,a}}{\cdot t}_{a,z}-\frac{x_{b}}{f_{x,b}}{\cdot t}_{b,z}\\ t_{a,y}=\frac{y_{a}}{f_{y,a}}{\cdot t}_{a,z}-\frac{y_{b}}{f_{y,b}}{\cdot t}_{b,z} \end{array}\right.$$

The above yields an initial 3-DoF translation, based on RGB images. However, when the depth information is considered, the object point cloud is applied to optimize the object pose. The point cloud of the target object is denoted as $P_{a}$ and its center of mass is represented by $\bar{P_{a}}$. Moreover, the nearest neighbor template point cloud is denoted as $P_{b}$ and its center of mass is $\bar{P_{b}}$; therefore, the *z*-axis displacement of the object can be obtained according to Equation (13):

(13) $${t_{a,z}}^{\prime }=\bar{P_{a}}-\bar{P_{b}}+t_{b,z}$$

Finally, the *x*-axis and *y*-axis displacements of the target object are recalculated according to Equation (12), and the final displacement pose of the object is represented using the following vector: $\left[t_{a,x},t_{a,y},{t_{a,z}}^{\prime }\right]$.

#### 3.5.3. The 6-DoF Object Pose Refinement

After obtaining the initial 6-DoF object pose, refinement is achieved by combining it with the ICP algorithm. Unlike the traditional ICP algorithm that directly performs fine alignment using the object point cloud and the source point cloud, this method is not very accurate since only part of the scene is visible. In this method, since the object rotation is generated as prior information, the coarse pose is applied to render the 3D model. This is combined with the high-precision object segmentation map, obtained from the instance segmentation algorithm, to generate the object point cloud from the depth image. Therefore, median filtering is applied to fill point cloud holes. To enhance alignment speed, voxel filtering and statistical filtering downsample the object point cloud, reducing the number of points while retaining the main information. Finally, the fine alignment is executed, resulting in the final 6-DoF object pose.

## 4. Experiment

For all experiments realized in this study, an Intel i7-12700k, GeForce RTX2080ti graphics processor, and 32GB RAM are used. For the software environment of this model, all models were implemented in Pytorch (v. 1.10), a Python version of Torch, Facebook’s open-source NN framework dedicated to GPU-accelerated NN programming.

Datasets: The initial T-LESS and LineMOD synthetic datasets are used for the study. They can be accessed using the following web link https://bop.felk.cvut.cz/datasets/ (accessed on 22 March 2023). As for the T-LESS dataset, it was also used in the study and is available at https://bop.felk.cvut.cz/datasets/#T-LESS (accessed on 12 January 2023). Data from 30 different weakly textured 3D models from the T-LESS dataset was employed. Moreover, the LineMOD dataset was used in the study, and it is available at BOP: Benchmark for 6D Object Pose Estimation (cvut.cz) (accessed on 12 October 2023).

Moreover, the MS COCO [42] dataset (available at https://cocodataset.org/ (accessed on 25 January 2023)) was employed in the training phase for pre-training of the M-ST network, and the Pascal VOC dataset (available at http://host.robots.ox.ac.uk/pascal/VOC/ (accessed on 25 January 2023)) was employed for the training of the CBAM–CDAE network while replacing the background image.

### 4.1. The 6-DoF Object Detection Results

#### 4.1.1. Training Detail

In this paper, the instance segmentation network, M-ST, undergoes pre-training on Microsoft COCO. The learning rate is initially set to 0.001 for 100 k iterations on a high-quality synthetic dataset obtained by secondary processing of synthetic data rendered by BlenderProc. The learning rate is then multiplied by 0.96 for every 1 k iterations in this paper. Where the bounding box information from target detection is cropped and adjusted before being input into the trained CBAM–CDAE.

The CBAM–CDAE network, benefiting from previous promising results, is trained using the DR method. Using OpenGL, we render 20 k views of each object uniformly at random 3D orientations and constant distance along the camera axis and resize to 128 × 128 × 3. We use the Adam optimizer with a learning rate of 0.0002. A batch size = 64 and about 40 k iterations. Details of the training process, along with the specific enhancement parameters, are elaborated in Table 1, respectively [25].

#### 4.1.2. Experiments on the LineMOD Dataset

We assess our method using the LineMOD dataset, a recognized benchmark for 6-DoF pose estimation of non-textured objects in cluttered scenes. The dataset comprises 13 objects and approximately 1200 RGB-D images per object. In our approach, we employed around 50 k high-quality synthetic images to train the M-ST network. The implementation of our method is conducted on pytorch. For example, in the instance segmentation network, we utilize SGD with momentum for optimization, employing a learning rate of 0.001, 40 k iterations, and a batch size of 256. On the other hand, for the CBAM–CDAE network, Adam was used for optimization, with a learning rate of 0.0002, 40 k iterations, and a batch size of 64.

In this paper, to showcase the effectiveness of our algorithm from various perspectives, we evaluate the performance of 13 objects in the LineMOD dataset using accuracy under the average distance difference (*ADD*) metric. Notably, the assessment involves training solely with synthetic data in the dataset, namely Benchvise, Cat, Duck, Holepuncher, Iron, Lamp, and Phone.

(14) $$ADD=\frac{1}{m}\sum_{M}\left\|\left(R_{x}+T\right)-\left(\tilde{R_{x}}+\tilde{T}\right)\right\|$$

For an indistinguishable view of the target, the average distance difference-symmetrical (*ADD-S*) is measured between the model vertices and their *ADD-S*.

(15) $$ADD\text{-}S=\frac{1}{m}\sum_{x_{1}\in M}\begin{array}{c} min\\ x_{2}\in M \end{array}\left\|\left(R_{x}+T\right)-\left(\tilde{R_{x}}+\tilde{T}\right)\right\|$$

Given the true values of the rotation matrix *R* and the translation matrix *T*, along with the estimated rotation matrix $\tilde{R}$ and the translation matrix $\tilde{T}$, *ADD* computes the average distances between the 3D model points for both sets. If the average distance between the true coordinates of the 3D mesh and the predicted pose estimate is less than 10% of the object diameter, the predicted pose is considered exact. Notably, in our test, LineMOD objects “eggbox” and “glue” are symmetric. Moreover, in this paper, we denote these two metrics as *ADD(-S)* and use the appropriate metric for the object.

Moreover, Table 2 presents the evaluation of all 13 objects in the LineMOD dataset using accuracy under the *ADD(-S)* metric. All considered methods are trained exclusively with synthetic data. Our method outperforms in detection accuracy for seven objects (Benchvise, Cam, Duck, Holepuncher, Iron, Lamp, and Phone); however, it does not lead for the symmetric objects “eggbox” and “glue”. Yet, the average accuracy across all objects is competitive with to the SyDPose, AAE, and 6IMPOSE methods, which are well based on synthetic data for positional pose estimation. In addition, our method is compared in the field of 6-DoF position estimation of objects based on synthetic data, and it is found to present certain advantages over today’s state-of-the-art algorithms on the LineMOD dataset.

#### 4.1.3. Experiments on the T-LESS Dataset

The T-LESS dataset comprises 20 scenes, providing an untextured CAD model and a textured 3D model for each object. Both models were created using three sensors to measure the texture of 30 common objects in industrial production, without distinct texture features and without distinguishing surface reflectance properties and colors. The dataset includes approximately 38,000 images for training, with each sensor contributing to both training and testing sets (10,000 images for testing). Training images feature a single example object against a black background, while test images showcase multiple example objects with a large range of colors, introducing clutter and heavy occlusion.

During the training process, 3D model views without 6-DoF object pose annotations were employed as training data. This approach falls under unsupervised learning, providing a cost-effective alternative to supervised learning. To quantify prediction results and measure recall for objects with or without textured surfaces, the visible surface difference (VSD) [43] metric was applied as represented here below.

(16) $$e_{VSD}\left(\tilde{D},D,\tilde{V},V,\tau \right)={avg}_{p\in \tilde{V}\cup V}\left\{\begin{array}{c} 0,p\in \tilde{V}\cap V\wedge \left|\tilde{D}\left(p\right)-D\left(p\right)\right|<\tau \\ 1,others \end{array}\right.$$

where $\tilde{D}$ represents the distance from the center of the camera to the 3D projection point obtained after estimating the object model, ${err}_{vsd}$ is determined by the distance between the estimated and ground truth visible object depth surfaces; moreover, in the test experiment, the thresholds τ=20 mm, θ=0.3 are set. 

Referring to Table 3, to visualize the results of our method through the T-LESS dataset, line plots methods were drawn to compare it with AAE [25], Pix2Pose [22] and Kehl et al. [44] method with RGB input and RGB-D input, respectively.

The results, displayed in Table 3, show that our method mostly dominates over the RGB and RGB-D input-based methods when considering the T-LESS dataset for 30 in textureless objects. The average recognition accuracy and recognition time are also displayed in Table 3.

Moreover, referring to Table 3, compared to the related work that uses only synthetic data for training, our method, whether based on RGB or RGB-D images, has shown a latency with respect to AAE [25], Pix2Pose [22], MP-AAE [26], and Xu et al. [40] methods in terms of detection speed, but outperformed these four methods in terms of detection accuracy. Although the recognition speed is not leading compared with some methods, the proposed method still meets the real-time requirements.

Moreover, Table 4 presents the average processing time of each stage of the algorithm, requiring 50 ms for object detection, and 230 ms for each object’s position estimation, leading to a total of 280 ms. Furthermore, this method requires 450 ms for position refinement combined with depth information. For applications with low real-time requirements, such as robot static grasping, the algorithms proposed in this paper meet the requests of practical applications.

However, to quantify the pose prediction results of the objects, all poses of the *n*th object in all scenes are predicted. In this work, we calculate the rotation error (RE) and the translation error (TE) of the object in the test set of the T-LESS dataset in the experiment. As a result, the error of estimated pose $\hat{P}=\left(\hat{R},\hat{t}\right)$ and the ground truth pose $\bar{P}=\left(\bar{R},\bar{t}\right)$ are measured using TE $\left(e_{TE}\right)$ and RE $\left(e_{RE}\right)$.

(17) $$e_{TE}\left(\hat{t},\bar{t}\right)={\left\|\bar{t}-\hat{t}\right\|}_{2}$$

(18) $$e_{RE}\left(\hat{R},\bar{R}\right)=arccos\left(\frac{Tr\left(\hat{R}{\bar{R}}^{-1}\right)-1}{2}\right)$$

In Equation (17), t is a 3 × 1 vector, and R is a rotation 3 × 3 matrix with an RE ranging between 0° and 180° as represented in Equation (18).

**Figure 10:**
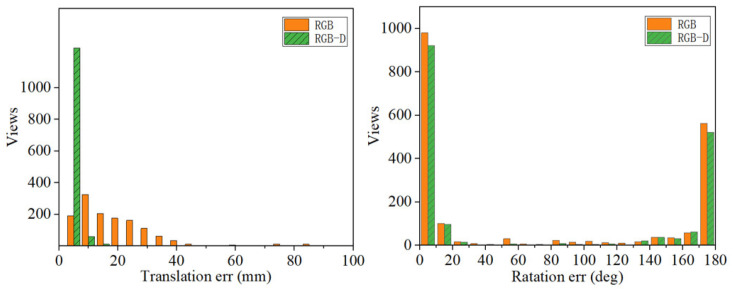
The histogram of rotation error for the 5th object, one view-dependent symmetry.

**Figure 11:**
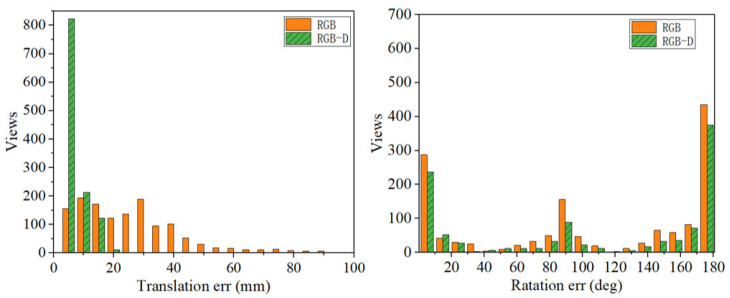
The histogram of rotation error for the 29th object, two view-dependent symmetry.

Concerning the histograms of error statistics proposed in Figure 10 and Figure 11, it is evident that both TE and RE are reduced when depth information is incorporated using RGB-D. The reduction in TE is particularly significant. It is evident that refining the pose, based on mask and depth information, has beneficial effects on the error reduction in the pose estimation task. 

**Figure 12:**
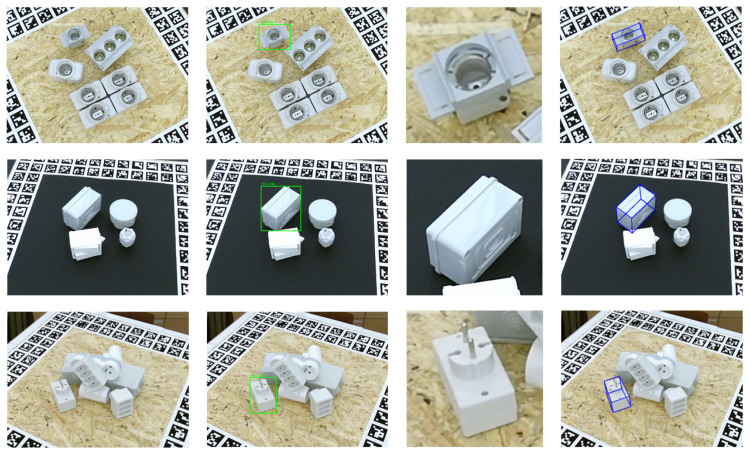
The 6-DoF pose visualization.

To visualize the detection process, as illustrated in Figure 12, the first column represents the image to be detected, whereas the second column is the result of target detection by the M-ST network and the third one denotes the cropped image with the network input shape. Finally, the fourth column represents the result of visualization.

#### 4.1.4. Analysis of Experimental Results

**Figure 13:**
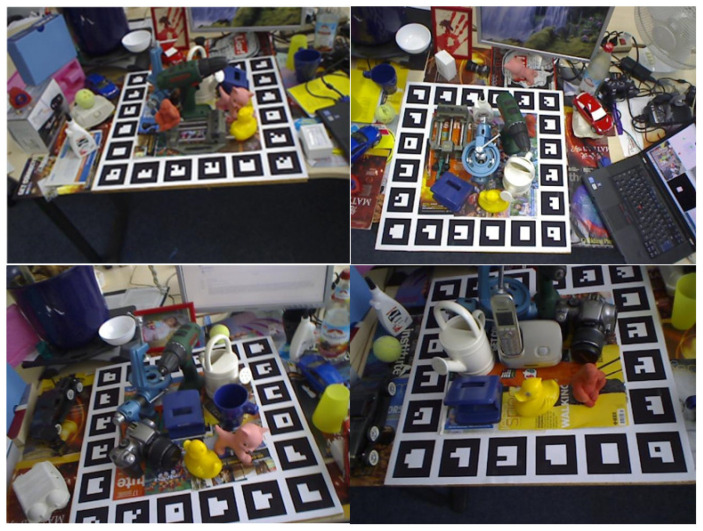
Schematic diagram of the LineMOD dataset.

**Figure 14:**
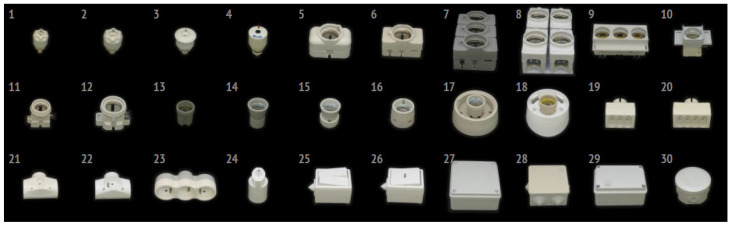
Schematic diagram of the T-LESS dataset.

In the paper, our method conducted a large number of experiments on T-LESS and LineMOD datasets (see Figure 13 and Figure 14). Comparing it to the same adopted synthetic data, we find that, compared with the current state-of-the-art algorithms, our method has shown a certain advantage in average recognition accuracy, and has achieved high recognition accuracy on both scenes when occlusion and weakly textured objects were considered. Meanwhile, we find that the recognition accuracy advantage is not obvious enough regarding symmetric objects, and the recognition speed of our method is not as fast as other methods when being tested over the T-LESS dataset. These findings also provide ideas for subsequent research work. 

### 4.2. Ablation Experiment on the T-LESS Dataset

#### 4.2.1. Results of M-ST

In this paper, to evaluate the recognition performance of the model when being applied to the T-LESS dataset, each detected picture in the target detection problem may contain multiple classes of target objects. Therefore, target detection has to find the objects contained in the picture, not only to classify the objects in the picture but also to localize them. As a result, target detection needs to evaluate the ability of the model to classify and localize objects for measuring the performance of the model.

(19) $$Precision=\frac{T_{P}}{T_{P}+F_{P}}$$

(20) $$Recall=\frac{T_{P}}{T_{P}+F_{N}}$$

(21) $$AP=\int_{0}^{1}p(r)dr$$

(22) $$mAP=\frac{\sum_{I=1}^{N}AP_{i}}{N}$$

In the above equations, *Precision* denotes the accuracy rate, *Recall* is the recall rate, $T_{P}$ represents the number of correctly detected samples $T_{N}$ indicates the number of correctly detected negative samples, $F_{P}$ is the number of incorrectly detected samples, and $F_{N}$ denotes the number of missed samples. In multi-target detection, a P-R curve can be plotted for each category. The average precision (AP) is the area under the P-R curve whereas the mean average precision (mAP) is obtained by calculating the average value of AP for multiple categories, as shown in Equations (21) and (22).

The experiments evaluated the mAP for the IoU when considering the 0.5 threshold cases. In addition, the experiments applied a number of parameters to measure the complexity of the model, and the ablation experiment results are displayed in Table 5.

Referring to Table 5, “Baseline” represents the training data that are generated by pasting objects from the T-LESS dataset with random translation, scaling, and in-plane rotation on random background images [26]. As for “Mask-RCNN + Swin”, it represents the Swin Transformer backbone network replacement operation for Mask-RCNN only, and “Mask-RCNN + iFPN” denotes the improvement of the FPN structure only, Finally, “Ours” denotes the M-ST instance segmentation network model used in this paper. 

By analyzing the results of the ablation experiments in Table 5, our improvements to the example segmentation network have yielded some effect, as the introduction of the Swin Transformer in the network reveals the most obvious effect, and the precision of “box” is higher than that of “seg”.

Moreover, the same ablation experiment as in Table 5 was validated on the LineMOD dataset, as shown in Table 6.

Upon analyzing the above experimental results, it is evident that the results of the instance segmentation network on the T-LESS dataset surpass that on the LineMOD dataset. This observation suggests that our method exhibits advantages when applied to weakly textured industrial objects.

#### 4.2.2. Results of 6-DoF Object Pose Estimation

To verify the effectiveness of each module of the proposed framework, ablation experiments were conducted. The overall framework was evaluated after being compared to the classical 6-DoF object pose estimation algorithm AAE network. Furthermore, the performance of the model was just compared with the CBAM module incorporated, the Swin Transformer algorithm incorporated, and finally, the proposed algorithm.

Table 7 shows the experimental results, where “Baseline” represents the CDAE combined with the Mask R-CNN instance segmentation network, the “CBAM-CDAE” denotes the result of improving only the CDAE network, “M-ST” indicates the result of improving only the Mask R-CNN network, and, finally, “OURS” represents the effect of the completed model proposed in this paper.

The results of the ablation experiments in Table 6 demonstrate that both the CBAM–CDAE network and the M-ST network contribute to the improvement of detection accuracy. The instance segmentation network exhibits a more pronounced effect on the improvement of the detection accuracy, revealing variations in the impact across datasets with different features.

## 5. Conclusions and Discussion

In this paper, we propose a DL-based 6-DoF object position estimation method relying on synthetic data. We leverage high-quality physically based rendering and DR aiming at addressing the domain gap between synthetic and real data in target detection and image segmentation tasks. Moreover, the proposed approach aims to overcome the challenges of low accuracy in current recognition based on synthetic data. This contribution holds significance for achieving high-precision pose estimation of weakly textured objects in various complex environments. Therefore, the advantages of this work can be summarized as follows:

1. In the example segmentation dataset processing, the BlenderProc realism dataset generation method, based on bilateral filtering processing, was employed to reduce the neighborhood problem between the synthetic data and the real data while obtaining a higher quality synthetic dataset;
2. In the network section, we introduce a Mask R-CNN network enhanced by the attention mechanism. This enhancement not only improves the accuracy but also reduces the number of model parameters. In addition, we propose an iFPN structure, addressing the deficiency in underlying feature information observed in the traditional FPN structure by adding a layer of bottom-up paths;
3. We also add a CBAM structure to the CDAE network to obtain a CDAE-CBAM network, yielding a better ability to extract potential feature vectors compared to the existent ones;
4. We put forth a CDAE-CBAM network, exhibiting enhanced potential feature vector extraction capabilities through the introduction of spatial and channel attention mechanisms compared to the pre-improvement one.

Our method not only improves the accuracy of pose estimation but also maintains a high detection speed. These findings contribute to the expansion and advancement of scientific research in the field of 6-DoF object pose estimation based on synthetic data. The experimental results demonstrate the effectiveness of the method and provide insights for future research in this area.

Future research will explore category-level 6-DoF object pose estimation tasks and lightweight networks with the aim of improving reliability and utility in industrial real-world scenarios.

## Figures and Tables

**Table 1:** The augmentation parameters of CBAM–CDAE.

	50% Chance (30% Per Channel)	Light (Random Position) and Geometric	
Add	*μ* (−0.1, 0.1)	Ambient	0.4
Contrast	*μ* (0.4, 2.3)	Diffuse	*μ* (0.7, 0.9)
Multiply	*μ* (0.6, 1.4)	Specular	*μ* (0.2, 0.4)
Invert		Scale	*μ* (0.8, 1.2)
Gaussian blur	*δ~μ* (0.0, 1.2)	Translation	*μ* (−0.15, 0.15)
		Occlusion	∈[0, 0.25]

**Table 2:** The accuracies of our method and the baseline methods on the LINEMOD dataset in terms of the *ADD(-S)* metric, where glue and eggbox are considered symmetric objects.

Method	AAE [25]	DPOD [18]	Ours (RGB)	SyDPose [32]	AAE [25]	SSD-6D [12]	6IMPOSE [27]	Ours (Depth)
Ape	3.96	37.22	23.7	16.4	20.6	65	78	77.6
Benchvise	20.92	66.76	45.2	35.2	64.3	80	92	**92.5**
Cam	30.47	24.22	55.6	16.7	63.2	78	66	**84.2**
Can	35.87	52.57	58.2	27.3	76.1	86	95	86.5
Cat	17.90	32.36	61.4	34.2	72	70	97	96.4
Driller	23.99	66.60	47.2	30.7	41.6	73	91	66.7
Duck	4.86	26.12	35.4	9.3	32.4	66	89	**92**
**Eggbox**	81.01	73.35	86.8	52.8	98.6	100	91	96.5
**Glue**	45.49	74.96	74.5	51.7	96.4	100	73	85.7
Holepunch	17.60	24.50	43.1	29.2	49.9	49	61	**88.3**
Iron	32.03	85.02	62.4	34.3	63.1	78	94	**95.4**
Lamp	60.47	57.26	72.5	37.5	91.7	73	87	**92.6**
Phone	33.79	29.08	56.4	17.2	71	79	74	**93.8**
Average	28.65	50.00	55.6	31.2	64.7	79	83.6	**88.3**

**Table 3:** The object recall for ${err}_{vsd}$.

Object	AAE [25] -RGB	Pix2Pose [22]	MP-AAE [26]	Xu et al. [40]	OURS -RGB	AAE [25] +Depth(ICP)	OURS- +Depth(ICP)
1	9.48	12.65	5.56	33.54	18.62	67.95	76.38
2	13.24	16.01	10.22	39.99	35.05	70.62	88.32
3	12.78	22.84	14.74	52.73	28.41	78.39	79.42
4	6.66	6.70	6.23	36.34	19.21	57.00	65.46
5	36.19	39.93	37.53	34.03	46.96	77.18	82.56
6	20.64	28.26	30.36	15.86	37.32	72.75	79.98
7	17.41	26.56	14.62	36.59	32.73	83.39	84.39
8	21.72	18.01	10.73	11.95	35.63	78.08	78.11
9	39.98	33.36	19.43	10.02	45.54	88.64	74.22
10	13.37	33.15	32.75	31.66	26.73	84.47	87.32
11	7.78	17.94	20.34	44.29	29.67	56.01	76.27
12	9.54	18.38	29.53	43.42	26.42	63.23	54.98
13	4.56	16.20	12.41	20.22	19.34	43.55	35.67
14	5.36	10.58	21.30	14.77	15.46	25.58	49.39
15	27.11	40.50	20.82	33.00	55.82	69.81	86.94
16	22.04	35.67	33.20	13.48	41.53	84.55	69.36
17	66.33	50.47	39.88	30.83	57.39	74.29	78.36
18	14.91	33.63	14.16	40.47	46.28	83.12	86.93
19	23.03	22.53	9.24	9.09	29.87	58.13	74.9
20	5.35	9.46	1.72	7.24	7.36	26.73	38.92
21	19.82	19.41	11.48	28.71	34.23	53.48	65.69
22	20.25	18.32	8.30	20.22	38.58	60.49	72.97
23	19.15	19.15	2.39	15.87	37.48	62.69	86.85
24	4.54	27.94	8.66	6.93	21.98	62.99	74.39
25	19.07	51.01	22.52	22.97	45.38	73.33	79.69
26	12.92	33.56	30.12	19.28	32.69	67.00	71.59
27	22.37	33.61	23.61	28.76	37.34	82.16	78.36
28	24.00	30.88	27.42	16.42	42.29	83.51	86.79
29	27.66	35.67	40.68	13.81	47.93	74.45	76.39
30	30.53	41.32	56.08	3.22	51.89	93.65	92.97
Mean (%)	19.26	26.79	20.53	**24.52**	**34.84**	68.57	**74.45**
Time (s)	**0.077**	0.127	0.2	/	0.18	**0.4**	0.63

**Table 4:** Processing time for each stage of the algorithm in this paper.

Stage Name	Average Processing Time (RGB)/ms	Average Processing Time (ICP)/ms
Object detection	50	50
Pose feature calculation	4	4
Similarity calculation	2	2
Translation calculation	124	124
ICP	/	450
Total time	180	630

**Table 5:** The mAP (%) for the example segmentation ablation experiment on the T-LESS dataset.

Model	Baseline	Mask R-CNN + Swin	Mask R-CNN + iFPN	Ours
box	68.9	75.2	72.1	78.2
seg	67.7	74.5	70.8	77.4

**Table 6:** The mAP (%) for the example segmentation ablation experiment on the LineMOD dataset.

Model	Baseline	Mask R-CNN + Swin	Mask R-CNN + iFPN	Ours
box	62.5	68.3	65.7	69.7
seg	61.2	66.8	63.9	67.8

**Table 7:** Results of ablation experiments for 6-DoF object attitude estimation on LineMOD dataset and TLESS dataset (ADD(-S) evaluation criterion is used for LineMOD dataset and VSD evaluation criterion is used for TLESS dataset).

Object	Baseline	CBAM–CDAE	M-ST	OURS
LineMOD	72.4	78.0	85.6	88.30
T-LESS	61.59	64.35	70.25	74.45

## Data Availability

For confidentiality reasons, no further details will be disclosed at this time.
